# Mental health status among family members of health care workers in Ningbo, China, during the coronavirus disease 2019 (COVID-19) outbreak: a cross-sectional study

**DOI:** 10.1186/s12888-020-02784-w

**Published:** 2020-07-17

**Authors:** Yuchen Ying, Liemin Ruan, Fanqian Kong, Binbin Zhu, Yunxin Ji, Zhongze Lou

**Affiliations:** 1 School of Medicine, Ningbo University, Ningbo, Zhejiang 315211 P.R. China; 2 Department of Psychosomatic Medicine, Ningbo First Hospital, Ningbo Hospital of Zhejiang University, 59 Liuting Street, Haishu District, Ningbo, Zhejiang 315211 P.R. China; 3 Ningbo College of Health Sciences, Ningbo, Zhejiang 315010 P.R. China; 4 Department of Medical Record and Statistics, Ningbo Medical Center Lihuili Hospital, Ningbo, Zhejiang 315041 P.R. China; 5 Department of Anesthesiology, The Affiliated Hospital of Medical School of Ningbo University, Ningbo, 315020 P.R. China

**Keywords:** Coronavirus disease 2019 (COVID-19), Mental health, Family members of health care workers, Anxiety symptoms, Depressive symptoms

## Abstract

**Background:**

To date, the psychological impact of COVID-19 epidemic among family members of health care workers (HCWs) in China has been neglected. This cross-sectional study investigates the mental health status and related factors in families of HCWs employed in designated hospitals in Ningbo, China.

**Methods:**

Family members of HCWs in five designated hospitals in Ningbo, China, were recruited in February, 2020 for this study. Demographic variables, COVID-19-related events in the lives of the participants, knowledge of COVID-19, and the working status of family members (that is, HCWs) were collected using online self-administered questionnaires. Mental health status was assessed using the Chinese versions of the Generalized Anxiety Disorder-7 (GAD-7) and Patient Health Questionnaire-9 (PHQ-9). Multivariable logistic regression analyses were performed to identify the main factors associated with the mental health conditions.

**Results:**

In total, 845 participants completed the questionnaires correctly (95.80% response rate). The prevalence of anxiety and depression symptoms were respectively 33.73% (95% CI: 30.53–36.92%) and 29.35% (95% CI: 26.27–32.43%) when a cut-off score of 5 was used for GAD-7 and PHQ-9. Risk factors for anxiety symptoms included more time (hours) spent thinking about the COVID-19, and whether or not family members (that is, HCWs) had direct contact with confirmed or suspected COVID-19 patients while high participants’ self-reported safety scores for HCW’s protective equipment was a protective factor. More time (hours) spent thinking about COVID-19, longer average working time per week worked by family members (that is, HCWs), and being parents and other next of kin of HCWs were risk factors for depressive symptoms. Compared to participants who were HCWs, participants who were private sector workers were more likely to develop depressive symptoms, while government or institutional employees were less likely to suffer from depressive symptoms.

**Conclusions:**

Psychological responses to COVID-19 have been dramatic among family members of HCWs during the rising phase of the outbreak. Our findings provide strong evidence to examine and attend to the mental health of this population during the COVID-19 epidemic.

## Background

In December 2019, a coronavirus disease 2019 (COVID-19) outbreak occurred in Wuhan, Hubei Province, China, which has spread rapidly throughout China and even the world [1]. As of May 31, 2020, a total of 83,017 COVID-19 confirmed cases with 4634 deaths had been reported in mainland China [2], and 5,934,936 confirmed cases with 367,166 deaths had been reported globally [3].

Due to the Chinese Lunar New Year holiday, more than 43,000 people traveled from Wuhan to Ningbo before the start of the travel ban on January 23 and the first confirmed case of COVID-19 in Ningbo was reported on January 21, 2020 [4]. To prevent and control the epidemic, the Ningbo government encouraged people to stay at home; discouraged mass gatherings; cancelled or postponed large public events; closed public places [5]; and set up 15 designated hospitals, of which five have treated for confirmed or suspected COVID-19 patients as of February 28, 2020 [6]. As a result of these vigorous policies, a total of only 157 COVID-19 cases had been confirmed in Ningbo as of February 21, 2020, and there have been no new cases in Ningbo since then [7]. Nevertheless, Ningbo had the fourth largest number of confirmed cases in East China in February [8].

Compelling evidence suggests that infectious disease pandemics, including the severe acute respiratory syndrome (SARS), the Middle East respiratory syndrome (MERS), and the 2009 novel influenza A (H1N1), were associated with mental health problems among the general population [9, 10], health care workers (HCWs) [11, 12], patients [13, 14], and family members of patients [15].

The severity of the COVID-19 outbreak in China is also causing mental health problems among HCWs such as stress, anxiety, and depressive symptoms [16]. One of the most important reasons for this could be that many HCWs lack contact with families [16]. They may also be afraid of the infection and of spreading the virus to their families [17]. Additionally, as most HCWs are shift workers, an occupation at higher risk for negative impact on family life, their families are demographically already particularly prone to mental health problems caused by family conflict [18]. Based on the above research data, we hypothesized that HCW families were likely to suffer from similar psychological problems during the COVID-19 epidemic, and that age, gender, educational level, occupation, nature of relationship with HCWs, COVID-19-related life events, participants’ knowledge of COVID-19 and the working status of family members (that is, HCWs) could be significant risk factors for mental health problems in HCW family members [19, 20].

However, the psychological response to the COVID-19 epidemic among HCW family members in China has been neglected. In the National Health Commission guidelines for emergency psychological crisis intervention for people affected by COVID-19, HCW family members were only ranked as the third priority group [21]. Moreover, while targeted mental health services have been provided to HCWs [22], children [23], and psychiatric inpatients in China [24], the mental health of HCW family members have remained largely neglected. In addition, to date, a series of studies have explored the psychological impact of COVID-19 outbreak on HCWs [19, 20], while no study has yet investigated the psychological impact of the COVID-19 epidemic among HCW family members.

To address this gap, the aim of the current study was to evaluate the mental health status of family members of HCWs and influencing factors who had direct or indirect contact with confirmed or suspected COVID-19 patients, and to provide data support to develop targeted interventions for this population to help them cope with psychological problems during the COVID-19 epidemic.

## Methods

### Study design

This was a cross-sectional study conducted in Ningbo in between February 10 and 20, 2020.

### Sample

A convenience sampling method was applied. First, with the help of the Health Commission of Ningbo, five designated hospitals in Ningbo that have treated confirmed or suspected COVID-19 patients were asked to participate in this survey. Second, we sent the online questionnaire to every department director of the five designated hospitals, which they then forwarded to every subordinate. Finally, HCWs forwarded the online questionnaire to their family members.

### Data collection

To prevent the spread of COVID-19 through droplets or contact, we used an online-based survey via the WeChat-based survey program “Questionnaire Star” to collect data.

### Participants

The inclusion criteria of this study were (1) being the next of kin of HCWs from hospitals designated for medical treatment of COVID-19 in Ningbo and (2) having access to the Internet. Exclusion criteria were as follows: (1) self-reported history of neurological disorders, mental illness, and other serious systemic disorders; and (2) self-reported substance abuse. All participants were informed of the study procedure and were invited to sign an informed consent form online upon their recruitment. Participants who passed the initial self-screening phase were asked to complete the self-administered questionnaires [25]. Of the 882 participants recruited for this study, 29 participants were excluded because they provided incomplete information, and eight were excluded because their family members took a vacation during the study period. Thus, 845 participants completed the survey. The response rate was 95.80%.

This study was approved by the Ethical Committee of Ningbo First Hospital, Ningbo, China (approval number: 2020-R042), and registered with the registry website http://www.chictr.org (registration number: ChiCTR2000030697).

### Measures of dependent variables

#### Depressive symptoms

We employed the Chinese version of PHQ-9 to assess the depressive symptoms of HCW families. PHQ-9 is a 9-item self-report measure to assess severity of depression. Participants rated each item in accordance with the frequency of symptoms over the past 2 weeks on a 4-point scale from 0 (not at all) to 3 (nearly every day). Total scores ranged from 0 to 27, with highest scores indicating greater severity of depressive symptoms [26]. The PHQ-9 has been widely used in China and good reliability and validity of the Chinese version of PHQ-9 has been demonstrated [27]. The Cronbach’s α was 0.92 in this study. The depressive symptom was defined as a total score of ≥5 points in the PHQ-9 according to the previous study during COVID-19 epidemic [28].

#### Anxiety symptoms

We employed the Chinese version of GAD-7 to assess the anxiety symptoms of HCW families. GAD-7 is a self-report questionnaire that screens and measures severity of generalized anxiety disorder. Participants rated seven items according to the frequency of symptoms in the past two weeks on a 4-point scale from 0 (not at all) to 3 (nearly every day). Total scores ranged from 0 to 21, with higher scores indicating greater severity of anxiety symptoms [29]. The GAD-7 has been widely used in China and good reliability and validity of the Chinese version of GAD-7 has been confirmed [30]. The Cronbach’s α was 0.92 in this study. The presence of anxiety symptoms was defined as a total score of ≥5 points in the GAD-7 according to the previous study during COVID-19 epidemic [28].

### Measures of independent variables

#### Demographics

The demographic characteristics included age, gender, educational level, occupation, and nature relationship with HCWs. Occupation included the following five types: (1) HCWs; (2) private sector workers; (3) government or institutional employees; (4) students; and (5) others, which consisted of freelancers, retirees, social workers, and other relevant staffs. The nature relationship with HCWs included the following four types: (1) spouses; (2) children; (3) parents; and (4) other next of kin.

#### Working status of family members (that is, HCWs), the participants’ knowledge of COVID-19, and COVID-19-related events in the lives of participants

The questionnaires for working status of family members (that is, HCWs), the participants’ knowledge of COVID-19, and COVID-19-related events in the lives of participants were self-developed specifically for this study, as there were no suitable scales available for measuring factors related to HCW families during the COVID-19 outbreak.

The working status of family members (that is, HCWs) included: (1) whether HCWs were in direct contact with confirmed or suspected COVID-19 patients; (2) the average working time (hours) per week for HCWs; (3) participants’ self-reported safety score for protective equipment of HCWs (safety scores ranging from 1 to 5, with higher scores indicating better protective effect); and (4) the department of HCWs, which was categorized into five types: (a) frontline departments, including the respiratory department, infectious diseases department, the intensive care unit, the fever clinic, and the isolation ward; (b) the medical technology department (imaging and laboratory departments, etc.); (c) the nursing department; (d) the logistics department; and (e) other departments.

Participants’ knowledge of COVID-19 was assessed based on responses to the following five COVID-19-related single-topic questions: (1) “Which of the following symptoms is not a common symptom of COVID-19?” with possible response options being fever, stuffy and runny nose, fatigue, and dry cough; (2) “how many days do people returning from Hubei Province need to be quarantined under observation?” with possible response options being 10 days, 12 days, 14 days, and 15 days; (3) “which of the following masks can prevent COVID-19?” with possible response options being activated carbon mask, cotton mask, sponge mask, and medical surgical mask; (4) “the known transmission routes of COVID-19 do not include which of the following?” with possible response options being contact transmission, droplet transmission, soil transmission, and aerosol transmission; and (5) “with regard to the disposal of discarded masks, what is incorrect about the following statement?” with possible response options being throw it away at any time when you run out of it; masks worn by people with fever need to be disinfected, sealed, and discarded; wash your hands immediately after handling the mask; and discarded masks should be discarded into hazardous trash cans. Of the above five questions, one point was given for each correct answer and no points were given for incorrect answers. A total score was calculated by summing points for each of the five questions, ranging from 0 to 5, with higher scores indicating a better knowledge of COVID-19.

COVID-19-related events in the lives of participants included: (1) whether there had been confirmed COVID-19 cases in families or friends; (2) whether there had been suspected COVID-19 cases in families or friends; and (3) time spent thinking about COVID-19, which was measured by the average number of hours spent thinking about the COVID-19 information every day.

### Statistical analysis

The Kolmogorov-Smirnov test was used to test the normal distribution of continuous data. Continuous variables were presented as mean ± standard deviation or medians (interquartile range [IQR]) depending on the distribution of the data. Categorical variables were presented as percentages. Univariate analysis was performed using the Student’s t-test or the Mann-Whitney U-test for continuous variables depending on the distribution of the data, and the chi-square tests for categorical variables.

Multivariate logistic regression analyses were used to assess the independent association of symptoms of depression and anxiety with independent variables. Variables with a *P* value of < 0.20 in univariate analysis were considered potential factors for inclusion into the multivariate logistic model [31], all these variables were entered into the final model since this was an exploratory study. Model discrimination and calibration were evaluated using C-statistic and Hosmer-Lemeshow goodness-of-fit statistic, respectively.

Statistical analyses were performed using SPSS v.22.0 (IBM Corp, Armonk, NY, USA). Two-sided *P* < 0.05 was considered statistically significant.

## Results

### Demographic characteristics

Table 1 presents characteristics of participants. The median age of participants was 37.00 years (IQR 32.00–44.00); 52.66% were male. Nearly half (46.98%) were also HCWs and 22.6% were enterprise workers; 65.44% were in a spousal relationship with the HCWs; and most of the participants (approximately 87.81%) had an education of junior college, bachelor’s degree, or above.

**Table 1:** Sample characteristics and univariate analysis of variables related to symptoms of anxiety and depression

Variables	n(%)/Median(IQR)	Anxiety symptoms (GAD-7 score)		***P***	Depressive symptoms (PHQ-9 score)		***P***
		< 5(*n* = 560)	≥5(*n* = 285)	< 5	< 5(*n* = 597)	≥5(*n* = 248)	
**Demographics**							
**Gender**				**0.042**			**0.012**
Male	445(52.66)	309(55.18)	136(47.72)		331(55.44)	114(45.97)	
Female	400(47.34)	251(44.82)	149(52.28)		266(44.56)	134(54.03)	
**Age(years)**	37.00(32.00–44.00)	36.00(31.25–43.00)	38.00(33.00–45.00)	**0.016**	36.00(32.00–43.00)	38.00(32.00–45.00)	**0.088**
**Educational level**				0.877			0.785
Secondary school or below	40(4.73)	26(4.64)	14(4.91)		29(4.86)	11(4.44)	
High school	63(7.46)	43(7.68)	20(7.02)		41(6.87)	22(8.87)	
Junior college or bachelor	650(76.92)	427(76.25)	223(78.25)		462(77.39)	188(75.81)	
Master or above	92(10.89)	64(11.43)	28(9.82)		65(10.89)	27(10.89)	
**Occupation**				0.340			**0.007**
HCWs	397(46.98)	259(46.25)	138(48.42)		279(46.73)	118(47.58)	
Private sector workers	191(22.60)	122(21.79)	69(24.21)		128(21.44)	63(25.40)	
Government employees or institutional employees	102(12.07)	74(13.21)	28(9.82)		84(14.07)	18(7.26)	
Students	21(2.49)	17(3.04)	4(1.40)		19(3.18)	2(0.81)	
Others	134(15.86)	88(15.71)	46(16.14)		87(14.57)	47(18.95)	
**Nature of relationship with HCWs**				**0.158**			**0.001**
Spouses	553(65.44)	365(65.18)	188(65.96)		405(67.84)	148(59.68)	
Children	40(4.73)	29(5.18)	11(3.86)		34(5.70)	6(2.42)	
Parents	49(5.80)	26(4.64)	23(8.07)		26(4.36)	23(9.27)	
Other next of kin	203(24.02)	140(25.00	63(22.11)		132(22.11)	71(28.63)	
**The COVID-19-related events in the lives of participants**							
**Whether there had been confirmed COVID-19 cases in families or friends**				0.264			0.879
Yes	3(0.36)	1(0.18)	2(0.70)		2(0.34)	1(0.40)	
No	842(99.64)	559(99.82)	283(99.30)		595(99.66)	247(99.60)	
**Whether there had been suspected COVID-19 cases in families or friends**				**0.186**			0.501
Yes	70(8.28)	41(7.32)	29(10.18)		47(7.87)	23(9.27)	
No	775(91.72)	519(92.68)	256(89.82)		550(92.13)	225(90.73)	
**Time to think about COVID-19 per day (hours)**				**0.006**			**0.018**
< 1	223(26.39)	160(28.57)	63(22.11)		160(26.80)	63(25.40)	
1–2	298(35.27)	203(36.25)	95(33.33)		222(37.19)	76(30.65)	
2–3	112(13.25)	77(13.75)	35(12.28)		83(13.99)	29(11.69)	
> 3	212(25.09)	120(21.43)	92(32.28)		132(22.11)	80(32.26)	
**Knowledge of COVID-19**	5.00(4.00–5.00)	5.00(4.00–5.00)	5.00(4.00–5.00)	0.653	5.00(4.00–5.00)	5.00(4.00–5.00)	0.348
**The working status of family members (that is, HCWs)**							
**The department of HCWs**				0.981			0.458
Front-line departments	131(15.5)	86(15.36)	45(15.79)		92(15.41)	39(15.73)	
Medical technology department	69(8.17)	46(8.21)	23(8.07)		50(8.38)	19(7.66)	
Nursing department	287(33.96)	192(34.29)	95(33.33)		213(35.68)	74(29.84)	
Logistics department	43(5.09)	30(5.36)	13(4.56)		28(4.69)	15(6.05)	
Other departments	315(37.28)	206(36.79)	109(38.25)		214(35.85)	101(40.73)	
**Whether HCWs directly contact with confirmed or suspected COVID-19 patients**				**0.019**			0.781
Yes	406(48.05)	253(45.18)	153(53.68)		285(47.74)	121(48.79)	
No	439(51.95)	307(54.82)	132(46.32)		312(52.26)	127(51.21)	
**The average working time per week worked by HCWs (hours)**	40.00(35.00–45.00)	40.00(35.00–45.00)	40.00(36.00–48.00)	**0.034**	40.00(35.00–44.00)	40.00(35.00–48.00)	**0.020**
**Participants’ self-reported safety score for protective equipment of HCWs**	4.00(3.00–5.00)	4.00(3.00–5.00)	4.00(3.00–5.00)	**0.004**	4.00(3.00–5.00)	4.00(3.00–5.00)	**0.178**

Numbers in bold indicate statistical significance at the 20% level

*Abbreviations*: *n* Number, *IQR* Interquartile range, *GAD* Generalized anxiety disorder, *PHQ* Patient Health Questionnaire, *HCWs* Health care workers, *COVID-19* 2019 Corona virus disease

Respectively 0.36 and 8.28% of participants had confirmed and suspected COVID-19 cases in families or friends. Most of the participants focused on the COVID-19 by spending more than 1 h every day (35.27% for 1–2 h, 13.25% for 2–3 h and 25.09% for 3 h or more, respectively.) The median score of participants’ knowledge of COVID-19 was 5.00 (IQR 4.00–5.00). Almost one sixth (15.5%) of the participants’ family members (that is, HCWs) worked in the front-line departments, while 33.96% worked in the nursing department and 37.28% worked in other departments; nearly half (48.05%) had direct contact with confirmed or suspected COVID-19 patients. The median working time per week worked by HCWs was 40.00 h (IQR 35.00–45.00), and the median points of participants’ self-reported safety score for protective equipment of HCWs was 4.00 (IQR 3.00–5.00).

### Univariate analysis of potential factors related to symptoms of anxiety and depression among HCW families during the COVID-19 epidemic

The prevalence of the symptoms of anxiety and depression and results of thee univariate analysis of potential factors related to anxiety and depression symptoms among HCW families are shown in Table 1.

The overall prevalence of anxiety and depression symptoms were 33.73% (95% CI: 30.53–36.92%) and 29.35% (95% CI: 26.27–32.43%), respectively.

Univariate analysis showed that gender, age, nature of relationship with HCWs, whether there had been suspected COVID-19 cases in families or friends, time spent thinking about COVID-19 per day (hours), whether HCWs had directly contacted with confirmed or suspected COVID-19 patients, average working time per week worked by HCWs (hours), and participants’ self-reported safety score for protective equipment of HCWs were potential factors related to anxiety symptoms, whereas educational level, occupation, whether there had been confirmed COVID-19 cases in families or friends, knowledge of COVID-19, the department where the HCW worked were not.

Univariate analysis also indicated that gender, age, occupation, nature of relationship with HCWs, time spent thinking about COVID-19 per day (hours), average working time per week worked by HCWs (hours) and participants’ self-reported safety score for protective equipment of HCWs were potential factors related to depressive symptoms, whereas educational level, whether there had been confirmed COVID-19 cases in families or friends, whether there had been suspected COVID-19 cases in families or friends, knowledge of COVID-19, the department where the HCW worked, whether or not HCWs were directly in contact with confirmed or suspected COVID-19 patients were not.

### Multivariate logistic regression analysis of factors significantly associated with anxiety and depression symptoms among HCW families during the COVID-19 epidemic

The association between influence factors with anxiety and depression symptoms among HCW families during the COVID-19 epidemic are given in Tables 2 and 3, respectively.

**Table 2:** Multivariate logistic regression analysis of variables related to anxiety symptoms

Variables	Anxiety symptoms (GAD-7 score ≥ 5)		
	*P*	*OR*	*95%CI*
**Gender**			
Male		Reference	
Female	0.052	1.370	0.998–1.880
**Age (years)**	0.127	1.013	0.996–1.031
**Nature of relationship with HCWs**	0.464		
Spouses		Reference	
Children	0.528	0.787	0.375–1.654
Parents	0.227	1.480	0.784–2.796
Other next of kin	0.502	0.877	0.597–1.288
**Whether there had been suspected COVID-19 cases in families or friends**			
No		Reference	
Yes	0.310	1.306	0.780–2.187
**Time to think about COVID-19 per day (hours)**	**0.006**	1.203	1.054–1.373
**Whether HCWs directly contact with confirmed or suspected COVID-19 patients**			
No		Reference	
Yes	**0.017**	1.440	1.067–1.944
**The average working time per week worked by HCWs**	0.110	1.009	0.998–1.020
**Participants’ self-reported safety score for protective equipment of HCWs**	**0.002**	0.810	0.707–0.928

Numbers in bold indicate statistical significance at the 5% level

*Abbreviations*: *n* Number, *GAD* Generalized anxiety disorder, *HCWs* Health care workers, *COVID-19* 2019 Corona virus disease, *OR* Odd ratio, *CI* Confidence interval

**Table 3:** Multivariate logistic regression analysis of variables related to depressive symptoms

Variables	Depressive symptoms (PHQ-9 score ≥ 5)		
	*P*	*OR*	*95%CI*
**Gender**			
Male		Reference	
Female	0.165	1.302	0.897–1.889
**Occupation**	**0.004**		
HCWs		Reference	
Private sector workers	**0.040**	1.579	1.021–2.441
Government employees or institutional employees	**0.045**	0.545	0.301–0.988
Students	0.220	0.362	0.071–1.838
Others	0.187	1.381	0.854–2.233
**Nature of relationship with HCWs**	0.017		
Spouses		Reference	
Children	0.388	0.649	0.243–1.734
Parents	**0.017**	2.243	1.156–4.355
Other next of kin	**0.032**	1.522	1.036–2.235
**Time to think about COVID-19 per day (hours)**	**0.010**	1.197	1.044–1.373
**The average working time per week worked by HCWs**	**0.006**	1.017	1.005–1.029
**Participants’ self-reported safety score for protective equipment of HCWs**	0.140	0.898	0.778–1.036

Numbers in bold indicate statistical significance at the 5% level

*Abbreviations*: *n* Number, *PHQ* Patient Health Questionnaire, *HCWs* Health care workers, *COVID-19* 2019 Corona Virus Disease, *OR* Odd ratio, *CI* Confidence interval

In multiple logistic regression analysis, participants who spent more time (hours) thinking about the COVID-19 (OR = 1.203, 95% CI: 1.054–1.373) and whose family members (that is, HCWs) had direct contact with confirmed or suspected COVID-19 patients (OR = 1.440, 95% CI: 1.067–1.944) were significantly more likely to develop anxiety symptoms, while higher participants’ self-reported safety score for protective equipment of HCWs (OR = 0.810, 95% CI: 0.707–0.928) was a significantly protective factor for participants to suffer anxiety symptoms. In addition, female participants were marginally significantly more likely to have anxiety symptoms (OR = 1.370, 95% CI: 0.998–1.880) than male participants. The final model showed good discrimination (C-statistic = 0.640, 95% CI: 0.601–0.678) and good calibration (χ^2^ = 5.906, degree of freedom = 8, *P* = 0.658).

Multiple logistic regression analysis also demonstrated that more time (hours) to focus on the COVID-19 (OR = 1.197, 95% CI: 1.044–1.373) and longer average working time per week worked by HCWs (OR = 1.017, 95% CI: 1.005–1.029) were significantly associated with a higher risk of depressive symptoms among participants. Compared to participants who were HCWs, private sector workers were significantly more likely to develop depressive symptoms (OR = 1.579, 95% CI: 1.021–2.441), while government or institutional employees (OR = 0.545, 95% CI: 0.301–0.988) were significantly less likely to have depressive symptoms. Compared to participants who are spouses of HCWs, parents (OR = 2.243, 95% CI: 1.156–4.355), and other next of kin (OR = 1.522, 95% CI: 1.036–2.235) were significantly likely to develop depressive symptoms. The final model showed good discrimination (C–statistic = 0.639, 95% CI: 0.599–0.680) and good calibration (χ^2^ = 4.902, degree of freedom = 8, *P* = 0.768).

## Discussion

This online-based cross-sectional study has provided evidence for the high prevalence of anxiety and depression symptoms among family members of HCWs in designated hospitals in Ningbo, China, during the COVID-19 epidemic. Therefore, as with HCWs, the mental health of HCW families need urgent attention.

We noted that 33.73% (95% CI: 30.53–36.92%) and 29.35% (95% CI: 26.27–32.43%) of family members of HCWs reported symptoms of anxiety and depression, respectively, which was much higher than the levels reported among the general population of China [32]. However, this prevalence was lower than those observed in a total of 1563 medical staff during the COVID-19 outbreak in a previous study using the same assessment instruments and cut-off scores as this study (44.7 and 50.7%, respectively) [28]. It is worth noting that this study was conducted in the early phase of the COVID-19 epidemic when most Chinese HCWs had been facing the most severe phase of the epidemic, likely to be causing extreme psychological responses [16]. Comparing our current study data with similar studies conducted at the same designated hospitals, it was interesting to note that family members of HCWs were more likely to develop symptoms of anxiety and depression than HCWs (28.8 and 23.9%, respectively). Although one needs to be cautious when comparing data from different studies using inconsistent time frames and medical conditions, this finding demonstrates, to some extent, an extreme psychological impact in association with the COVID-19 epidemic in family members of HCWs.

Studies have demonstrated that hospital-related transmission was suspected to be the possible mechanism of infection for affected HCWs [33]. Thus, it is easy to understand that family members (that is, HCWs), who had direct contact with confirmed or suspected COVID-19 patients, were associated with higher risk for anxiety symptoms among participants because they were excessively concerned that their families might be infected or even die. This is consistent with the results of a previous study that reported that family members of H1N1 patients suffer from anxiety [15]. Moreover, without proper personal protective equipment, COVID-19 may endanger HCWs [34]. As a result, it is understandable that participants who report a higher safety score for protective equipment of HCWs would believe that their families were better protected, and therefore, they were less likely to develop anxiety symptoms.

Our findings demonstrated that longer average working time per week worked by HCWs was significantly associated with a higher risk of depressive symptoms among participants. Longer working time means that HCWs have to spend more time in contact with confirmed or suspected COVID-19 patients, which may increase their chances of being infected with COVID-19 [33], and cause depressive symptoms in family members [15]. Longer working hours are also more likely to eat into the family time of HCWs, and may therefore cause work-family conflict between HCWs and their family members, resulting in depression among both HCWs and their family members when coping with the conflict [35]. It is interesting to note that, compared to participants who were HCWs, enterprise workers were more likely to develop depressive symptoms. Most enterprise workers have no medical background and may thus lack sufficient knowledge about the COVID-19 outbreak. They may develop depressive symptoms because they may lack the psychological endurance necessary for a pandemic [36]. In addition, many enterprises were forced to shut down production during the epidemic in China, resulting in the loss of income for their employees. However, loss of income as a predicting factor had the highest correlation with depression among enterprise workers, according to the results of a previous study conducted during the 2003 SARS outbreak [37]. Government or institutional employees were less likely to have depressive symptoms, this has been less documented in the literature. It is possible that this population were more likely to have access to clear and unambiguous information on the epidemic, so they are less likely to suffer from feelings of uncertainty, which is a known risk factor for depression during an infectious epidemic [38]. Moreover, compared to HCWs who suffer from depression during an infectious epidemic, including the COVID-19 outbreak [19, 20], most government or institutional employees stayed away from confirmed or suspected COVID-19 patients, and therefore had a lower risk of depressive symptoms than HCWs. Similar to the results of a previous study on H1N1 carried out in 2009, higher depression was noted among those in non-spousal relationships with HCWs, i.e., in our sample, these were parents and other next of kin [15]. Considering the majority of parents of HCWs were already elderly, one possible explanation is that the rapid transmission of COVID-19 and high death rate may have exacerbated the risk of mental health problems and worsened existing psychiatric symptoms among older adults [39]. We also speculate that most parents and other next of kin of HCWs have no medical background, and may therefore have a more extreme psychological response to COVID-19 epidemic, as previously discussed [36].

The only factor significantly associated with symptoms of both anxiety and depression was the time spent thinking about COVID-19, which was consistent with findings in the general population during the COVID-19 outbreak [38]. During this period of COVID-19 outbreak, most of the general population, including family members of HCWs, were isolated at home and received a great deal of information, as the government ran national messaging campaigns that constantly emphasized the dangers of COVID-19, especially for affected HCWs. Thus, family members of HCWs had more time to gather information through the Internet and the media about HCWs who treated COVID-19 patients [40], for example on WeChat, which had a wider psychological impact, such as symptoms of anxiety and depression, among the public [17]. Moreover, the expression of this psychological reaction may be the normal protective response of the human body to the pressure of the epidemic; this also occurred during the SARS outbreak in 2003 [41].

Our study findings indicate that insufficient and inadequate attention is being paid to family members of HCWs during the COVID-19 epidemic in China. Given the increasingly serious outbreak outside of China, the main implication of this study is that our findings could assist in developing mechanisms to help family members of HCWs in similar situations in other countries. In order to alleviate symptoms of anxiety and depression among this vulnerable population, several appropriate interventions are recommended as follows: first, health policy makers and stakeholders should collaborate to provide high-quality, timely crisis psychological services to HCW families. Online psychological self-help intervention systems, including online cognitive behavioral therapy for depression and anxiety (e.g., on WeChat), would be appropriate for HCW families [28]. Second, providing suitable protective equipment, work schedules, and accommodation to HCWs would benefit family members who are concerned about HCWs being infected. Third, according to the findings of previous studies [42], social support appeared to have a protective effect on mental health problems due to possible conflicts between HCWs and their families. Thus, we strongly recommend both sides to take the initiative to communicate with each other to show their support. Finally, the government’s propaganda strategies should be well-organized and effective [43]. The provision of reliable and transparent epidemic information to family members of HCWs is essential to enhance their sense of control and self-efficacy so they are able to cope with the psychological impact of the COVID-19 outbreak [44].

This study has some important strengths. First, to the best of our knowledge, this is the first study to explore the mental health problems and related factors among family members of HCWs during the COVID-19 outbreak and one of the first to investigate this issue during an infectious epidemic. Second, mental health status was assessed using previously developed measurement tools with good reliability and validity. Third, the use of multivariate analysis may cause type I error inflation.

Nevertheless, several limitations need to be considered. First, because we performed a cross-sectional study, our results do not show a causal relationship. Second, due to the sudden outbreak of the epidemic, baseline data on mental health conditions in the target population during normal conditions without an outbreak were not available so that excess morbidity in terms of anxiety and depression symptoms due to the outbreak cannot be correctly determined. Thus, we compared the prevalence of depression and anxiety symptoms among target populations with that observed in the general population in China during the normal conditions, as we discussed above. Third, to prevent potential COVID-19 infection from spreading, a web-based survey was conducted; study sampling was therefore voluntary, resulting in possible selection bias. Fourth, our sample is not highly representative, as the respondents were all from Ningbo City.

## Conclusions

The present study provided evidence of a major mental health burden of family members of HCWs in designated hospitals during the COVID-19 epidemic in Ningbo, China, particularly in participants who spent more time thinking about COVID-19, family members (that is, HCWs) who had direct contact with confirmed or suspected COVID-19 patients, family members (that is, HCWs) with longer average working time per week, and those who were in non-spousal relationships with HCWs. On the contrary, participants’ high self-reported safety score for protective equipment of HCWs was a protective factor against psychological problems. Compared to participants who were HCWs, those who were enterprise workers were more likely to develop mental health problems, while those who were government or institutional employees were less likely to report psychological issues. In summary, we suggest that more attention should be paid to the mental health of this vulnerable population during an infectious disease outbreak. In addition, our findings are important in enabling the government to allocate health resources and offer appropriate treatment for family members of HCWs who suffer mental health problems during the COVID-19 epidemic or any other infectious disease outbreak in the future.

## Data Availability

The datasets obtained and/or analyzed during the current study are available from the corresponding author on reasonable request.

## Abbreviations

HCWs: Healthcare workers
COVID-19: Coronavirus disease 2019
SARS: Severe acute respiratory syndrome
MERS: Middle East respiratory syndrome
H1N1: 2009 novel influenza A
GAD-7: Generalized Anxiety Disorder-7
PHQ-9: Patient Health Questionnaire-9
IQR: Interquartile range
