# Correction to: Mental health status among family members of health care workers in Ningbo, China, during the coronavirus disease 2019 (COVID-19) outbreak: a cross-sectional study

**DOI:** 10.1186/s12888-021-03048-x

**Published:** 2021-01-25

**Authors:** Yuchen Ying, Liemin Ruan, Fanqian Kong, Binbin Zhu, Yunxin Ji, Zhongze Lou

**Affiliations:** 1 School of Medicine, Ningbo University, Ningbo, Zhejiang 315211 P.R. China; 2 Ningbo College of Health Sciences, Ningbo, Zhejiang 315010 P.R. China; 3 Department of Psychosomatic Medicine, Ningbo First Hospital, Ningbo Hospital of Zhejiang University, 59 Liuting Street, Haishu District, Ningbo, Zhejiang 315211 P.R. China; 4 Department of Medical Record and Statistics, Ningbo Medical Center Lihuili Hospital, Ningbo, Zhejiang 315041 P.R. China; 5 Department of Anesthesiology, The Affiliated Hospital of Medical School of Ningbo University, Ningbo, 315020 P.R. China

**Correction to: BMC Psychiatry 20, 379 (2020)**

**https://doi.org/10.1186/s12888-020-02784-w**

Following the publication of the original article [1], the authors identified a mismatch between the assigned affiliations and numbering. The correct assigned affiliations and numbering are given below.

Yuchen Ying1,2,3†, Liemin Ruan3†, Fanqian Kong4, Binbin Zhu5, Yunxin Ji3 and Zhongze Lou3*

2. Ningbo College of Health Sciences, Ningbo, Zhejiang 315,010, P.R. China.

3. Department of Psychosomatic Medicine, Ningbo First Hospital, Ningbo Hospital of Zhejiang University, 59 Liuting Street, Haishu District, Ningbo, Zhejiang 315,211, P.R. China.

The author group has been updated above and the original article [1] has been corrected.
